# Contribution of Phosphorylation Modification to Stability and Antibacterial Activity of Egg White Protein Nanogels Loaded with Cinnamon Bark Essential Oil

**DOI:** 10.3390/gels11010012

**Published:** 2024-12-27

**Authors:** Sheng-Qi Rao, Xin-Ru Gao, Hui Liu, Zhi-Rong Wang, Zhen-Quan Yang

**Affiliations:** 1 College of Food Science and Engineering, Yangzhou University, Yangzhou 225127, China; rsq2001@163.com (S.-Q.R.); wfy2025@163.com (H.L.);; 2 Key Laboratory of Catering Food Processing and Safety Control (Yangzhou University), China General Chamber of Commerce, Beijing 100711, China; 3 Jiangsu Key Laboratory of Zoonosis, Yangzhou 225009, China

**Keywords:** cinnamon bark essential oil, phosphorylation, egg white protein, nanogels, antibacterial properties

## Abstract

This study evaluated the potential usage of phosphorylated egg white protein (P-EWP) nanogels fabricated via microwave-induced phosphorylation modification and gel process and further ultrasonic nanometrization as novel delivery systems for cinnamon bark essential oil (CBEO). Compared to EWP-CBEO nanogels without chemical phosphorylation, the obtained P-EWP-CBEO nanogels have shown smaller average hydrodynamic diameter (133.6 nm), relatively uniform size distribution (polydispersity index around 0.265), enhanced negative surface charge (−35.4 mV), and improved stability under the conditions of high temperature (up to 90 °C) and ionic strength (up to 200 mM NaCl). Moreover, P-EWP-CBEO nanogels, with hydrophobic interactions and disulfide bonds as the main intermolecular forces, exhibited a remarkable conformational change in microstructures. In addition, the results of the antibacterial experiments on *Escherichia coli*, *Staphylococcus aureus*, and *Listeria monocytogenes* showed that the MIC values of P-EWP-CBEO nanogels were two times lower than those of EWP-CBEO nanogels and could completely inhibit the growth of pathogenic bacteria within 108 h. Hence, we have suggested that P-EWP-CBEO nanogels are successfully fabricated with improved physicochemical properties as novel potential natural preservatives in the food industry.

## 1. Introduction

Foodborne disease is a major public health problem around the world. Although using chemical food preservatives is one of the effective strategies for controlling pathogenic bacteria, many studies have shown that improper use of them can produce certain side effects [1]. Recently, natural antibacterial agents have attracted growing attention from all kinds of audiences due to their safety, effectiveness, and environmental friendliness [2]. Among these, essential oils (EOs), natural oily liquids originating from flowers, leaves, fruits, stems, stem bark, and other parts of plants, are widely considered to be one of the most promising substitutes for a range of chemical agents [3]. A particularly notable example is cinnamon bark essential oil (CBEO), which has been proven to exhibit strong antioxidant and antibacterial activities owing to high contents of eugenol and cinnamaldehyde [4]. Ample evidence indicates that CBEO is effective against both Gram-negative and Gram-positive bacteria, especially efficient for pathogenic bacteria, including *Salmonella typhi*, *Pseudomonas aeruginosa*, *Listeria monocytogenes*, *Escherichia coli* O157:H7, *Staphylococcus aureus*, etc. [5]. More importantly, CBEOs and their purified constituents are generally recognized as safe (GRAS) by the Food and Drug Administration [6]. 

The volatile property, low water solubility, and high sensitivity to the environment of EOs result in reduced and transient contact with pathogenic bacteria in foods that generally contain high moisture content [7]. This limits its large-scale commercial and industrial applications and can potentially be overcome by covalent encapsulation. Of these, the last several decades have witnessed global research insights into nanoencapsulation, which is defined as a technology to pack substances in miniature, making use of techniques such as nanocomposite, nanoemulsification, and nanoestructuration [8]. Thus, nanoencapsulation can significantly enhance the stability of EOs and retain and even strengthen their antimicrobial activities by protecting the enterprising ingredient and controlling the abiding release of the active components [9]. For example, Shen suggested nanoencapsulation mainly contributes to the improved compatibility between clove EO-loaded nanocarriers and pullulate-gelatin produced by the Tween 80 and whey protein isolate/inulin mixture [10]. Similarly, our previous study showed that the gel-embedding encapsulation of carvacrol, the predominant compound in oregano EO, in ovalbumin at pH 2 greatly enhances its antibacterial activity against *Bacillus cereus* and *Salmonella* [11].

In the last decades, protein-based nanogels have been widely used for encapsulation and delivery of bioactive agents due to high encapsulation efficiency, favorable biocompatibility, and controlled release potential/efficacy [12]. Nanogels are in the range of 1–1000 nm with a three-dimensional network structure, which is formed by chemical or physical cross-linking of polymers with hydrophilic or amphiphilic macromolecular chains, typically featuring the characters of hydrogels and nanoparticles at the same time [13]. Compared with whey and rapeseed proteins, which are widely used as efficient vectors in food, notably, egg white protein (EWP) is more capable of forming gels or fabricating various nanomaterials because its main components are ovalbumin (54%), oval transferrin (12–13.6%), oval mucin (11%), and lysozyme (3.4–3.5%), all of which are globulins [14]. In spite of this, nanogels formulated with proteins are highly susceptible to solvent conditions, such as pH, ionic strength, and temperature, which have a great influence on protein aggregation state and kinetics. Worse yet, the relatively weak emulsification of EWPs cannot be effectively combined with hydrophobic substances, creating an obstacle to the application of EWPs-EOs.

Many application defects of protein-based nanogels can be addressed through chemical modification of protein, including glycosylation, acid and alkali hydrolysis, phosphorylation, acylation, etc. [15]. Phosphorylation, with the characteristics of low cost, extensive application range, and remarkable effect, has been widely regarded as an excellent method for modifying food proteins. In particular, sodium tripolyphosphate (STP) is a polyionic crosslinking agent with three phosphate groups that was used to change and enhance the properties of chitosan. Phosphorylation can increase electronegativity among protein molecules and reduce the isoelectric point (pI) of the proteins, thereby improving the solubility, emulsification, foaming, and protein gelling ability [16]. Yang showed that STP significantly reduces the gliadin nanoparticle size and enhances the storage and temperature stability of gliadin and zein nanoparticles through the introduction of phosphate groups [17]. It is worth noting that protein phosphorylation can improve electron conduction and adhesion, leading to a remarkable increase in the stability and delivery efficiency of many bioactive compounds [18]. Microwave-assisted technology can significantly shorten the reaction time. Wang assisted phosphorylated soy protein isolate under 600 W microwave for 3 min. The results showed that the emulsifying activity of the phosphorylated protein was 2 times higher and the emulsifying stability was 1.4 times higher [19].

Therefore, inspired by this open question and informed by our previous work, the aim of this study was to prepare and characterize the phosphorylated EWP loaded with CBEO (P-EWP-CBEO) nanogels, which were consequently compared with CBEO and EWP-CBEO nanogels in terms of thermal and ionic strength stability and long-term antibacterial activities against *E. coli*, *S. aureus*, and *L. monocytogenes*.

## 2. Results and Discussion

Therefore, after optimizing the oil-to-wall ratio, pH value, and protein concentration, when the oil-to-wall ratio was 1:7, pH = 8, and the protein concentration was 6.3%, the final encapsulation efficiency was 93.79 ± 1.933%.

### 2.1. Preparation of P-EWP-CBEO

**Figure 1:**
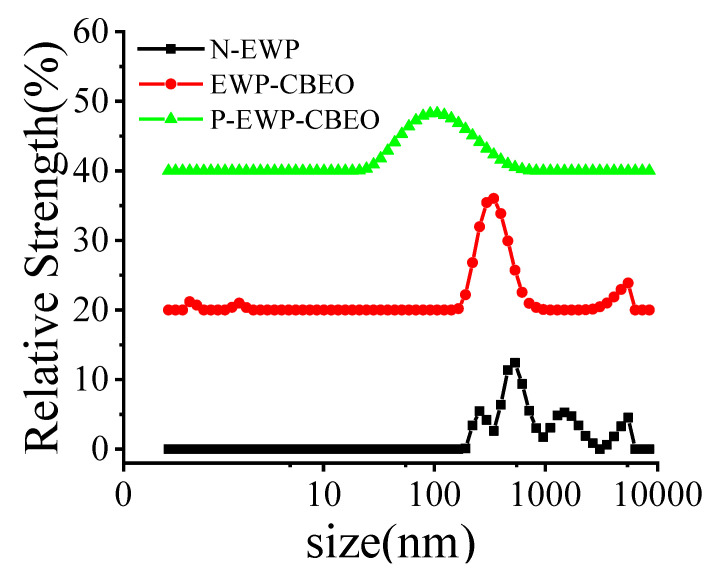
Particle size distribution of P-EWP-CBEO nanogels.

The average particle size and PDI of EWP-CBEO and P-EWP-CBEO nanogels are shown in Table 1 and Figure 1. The average particle size of EWP-CBEO nanogels significantly decreased (*p* < 0.05) from 378.8 nm to 133.55 nm after phosphorylation. This reduction may be attributed to enhanced repulsive forces during phosphorylation, which can inhibit the aggregation of droplets to each other. PDI is used to evaluate the homogeneity of the sample solution. The higher the value, the wider the particle size distribution. The PDI of P-EWP-CBEO nanogels was 0.265, which was remarkably lower than that of EWP-CBEO nanogels (0.418) [20]. It appears that phosphate groups negatively charge the surface of nanogels, enhancing their charge strength, which may increase the repulsive force between nanogels. Therefore, phosphorylated protein nanogels with smaller particle sizes and lower PDI are more stable because their enhanced charge prevents aggregate formation and improves their stability [21]. Compared to EWP-CBEO nanogels, the EE of P-EWP-CBEO nanogels increased from 78.34 ± 2.88% to 93.79 ± 1.933%.

### 2.2. Intermolecular Forces That Maintain Nanoparticle Structure

The interconversion of intermolecular forces (electrostatic interactions, hydrogen bonds, disulfide bonds, and hydrophobic interactions) is generally considered to be one of the important reasons for the formation of protein complexes. It has been observed previously that hydrogen bonds cleave, hydrophobic groups expose, and disulfide bonds form during thermal denaturation of EWP [22]. NaCl, DTT, urea, and SDS are protein denaturants with the ability to disrupt electrostatic interactions, disulfide bonds, hydrogen bonds, and hydrophobic interactions [23]. As shown in Supplement Figure S1, after adding the denaturant, NaCl increases the particle size of EWP-CBEO nanogels. Urea and DTT do not change the particle size of EWP-CBEO nanogels, and SDS can significantly reduce the particle size of EWP-CBEO nanogels from 378.8 ± 28.3 nm to 172.6 ± 3.85 nm. The results showed that the main reasons for the maintenance of the microstructure of EWP-CBEO nanogels were hydrophobic interactions. After adding denaturant to P-EWP-CBEO nanogels, the particle size of P-EWP-CBEO nanogels does not change significantly after adding urea, and the particle size of the sample increases after the addition of NaCl, while DTT and SDS significantly reduce the particle size of the samples, indicating that disulfide bonds and hydrophobic interactions are the main intermolecular forces maintaining P-EWP-CBEO nanogels. A small number of disulfide bonds were detected, which may be the result of the interaction with ovalbumin and lysozyme contained in the reported egg white protein, and the sulfhydryl groups in ovalbumin and lysozyme are easily converted to disulfide under the induction of external heating bonds, resulting in instability of the colloidal structure and leading to aggregation. A previous study showed that the hydrophobic interaction of phosphate-treated gels could significantly enhance this phenomenon with increasing heating time [24]. P-EWP-CBEO intermolecular forces in the figure have more hydrophobic interactions because of their more stretched three-dimensional structure and exposure to hydrophobic groups. An appropriate intermolecular force ratio is beneficial to the stability of protein complex structure.

### 2.3. Structural Characterization of P-EWP-CBEO

#### 2.3.1. FTIR Analysis

**Figure 2:**
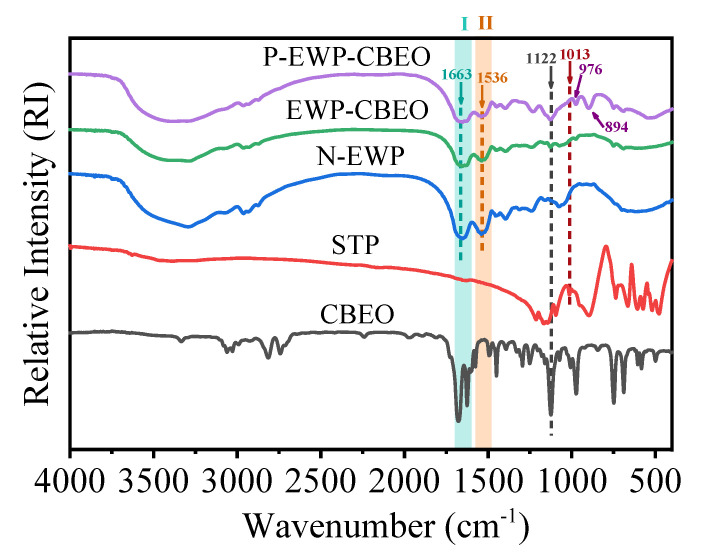
FTIR spectra of P-EWP-CBEO nanogels.

The results of N-EWP (control group as measured with untreated EWP powder), EWP-CBEO, and P-EWP-CBEO nanogels are shown in Figure 2. FTIR spectrometry gives useful information concerning the protein’s secondary structure. Proteins have many characteristic absorption bands in the infrared region, such as amide I (1600–1700 cm^−1^) and amide II (1480–1575 cm^−1^), which are primarily responsible for secondary structure [25]. The peak at 1663 cm^−1^ is related to the stretching vibration of -C=O forming -CONH-, and the peak at 1536 cm^−1^ corresponds to the coupled vibrations of N-H bends and C-N stretches in amide absorption band II. Compared with N-EWP, the wave numbers of EWP-CBEO and P-EWP-CBEO nanogels were slightly lower, indicating that gel parameterization and phosphorylation modification affect the secondary structure of the protein. When P-EWP-CBEO was phosphorylated, the peak was attributed to the P-O stretching vibration at 1122 cm^−1^, and the phosphate stretched asymmetrically at 1013 cm^−1^, which proved that STP was successfully attached to EWP. In addition, P-EWP-CBEO nanogels showed new absorption peaks at 976 cm^−1^ and 894 cm^−1^, which belong to P-O stretching and P=O stretching, respectively. The results are consistent with a study [26], which indicated that the protein phosphate groups are successfully introduced into the side chains of protein nanoparticles. 

#### 2.3.2. DSC Analysis

**Figure 3:**
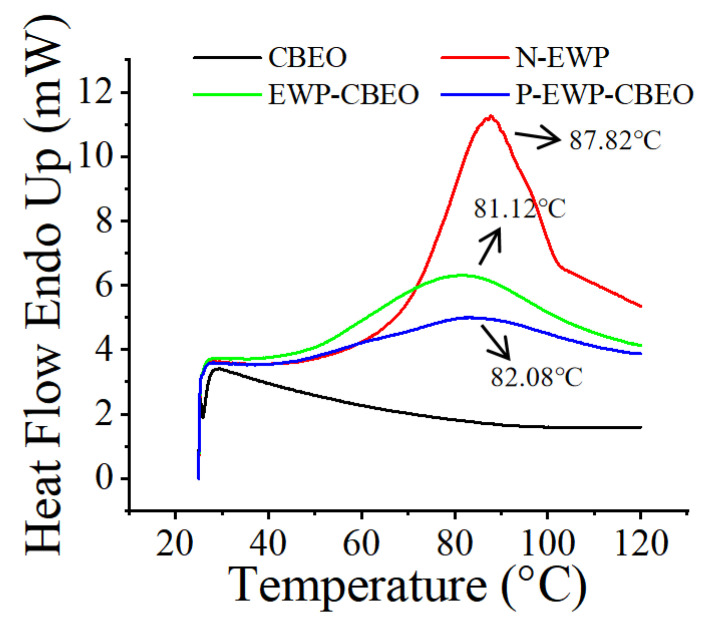
Differential scanning thermal analysis of P-EWP-CBEO nanogels.

Differential scanning calorimetry (DSC) is extensively used for the characterization of protein stability, for comparisons between native and mutant variants of proteins, and optimization of buffer conditions [27]. Figure 3 shows the DSC curves of EWP, EWP-CBEO, and P-EWP-CBEO nanogels in DSC during heating. N-EWP showed a heat-absorbing peak at about 87.82 °C, indicating that the water evaporates from N-EWP during heating, consistent with previous reports [28]. The absorption peak of EWP-CBEO nanogels was observed at 80–90 °C, which corresponded to the melting and decomposition of EWP and also confirmed the formation of an inclusion complex between EWP and CBEO. As shown in Table 2, N-EWP and EWP-CBEO exhibit T*_p_* values of 87.82 °C and 81.12 °C, respectively, suggesting that STP incorporation lowers protein thermal stability, likely due to its impact on the hydrogen bonding, hydrophobic interactions, and van der Waals forces of proteins, hence altering thermal stability. Compared with EWP-CBEO nanogels, the DSC peak of P-EWP-CBEO nanogels changed significantly and tended to be gentle. Therefore, these characteristics helped us determine that STPP was successfully linked to EWP, resulting in a decrease in its phase transition temperature and improved thermal stability. Our results were consistent with the previous reports [29].

#### 2.3.3. SEM Observation

**Figure 4:**
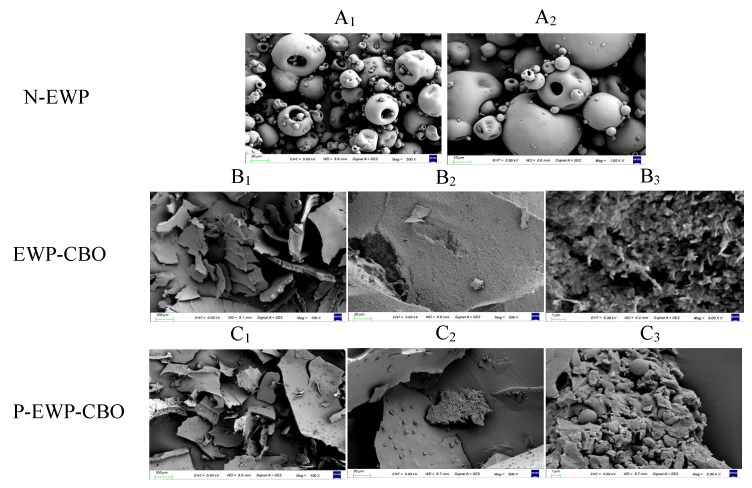
Scanning electron micrography (SEM) of freeze-drying egg white protein powder (**A1**,**A2**), egg white protein-embedded cinnamon essential oil nanogels (EWP-CBEO) (**B1**–**B3**), and phosphorylation-modified egg white protein-embedded cinnamon essential oil nanogels (P-EWP-CBEO) (**C1**–**C3**).

The microstructural changes of protein gel nanoparticles were explained by SEM observation at 100 × (Figure 4A1,B1,C1), 500 × (Figure 4A2,B2,C2), and 1000 × (Figure 4B3,C3) magnifications. N-EWP exhibited loose and porous microspheres, and the size of the microspheres and particles was not uniform. The previous study by Han et al. also reported that the reduction in pore size reflected the increased interconnectivity of the network structure [30]. After microwave phosphorylation of EWP into nanogels, it showed a sheet-like morphology, and EWP-CBEO and P-EWP-CBEO nanogels exhibited some relatively compact structures with some unevenly distributed pores of different sizes. Compared with N-EWP, the morphology of EWP-CBEO and P-EWP-CBEO nanogels was very different, and the gel network structure became finer and denser, with a large number of needle-like aggregates of different sizes, which may be caused by microwave heating and ultrasonic treatment of EWP powder [28]. A large number of fine cracks and fragments were observed in the lamellar aggregates of P-EWP-CBEO nanogels, indicating that the phosphorylation treatment has disrupted the otherwise dense aggregation state. This phenomenon may be due to the introduction of phosphate groups, which increases the charge repulsion between proteins, resulting in proteins that are less prone to aggregation [31]. However, compared with EWP-CBEO, P-EWP-CBEO nanogels formed many smaller microspheres, suggesting that phosphates may be cross-linked with EWP to form finer and more ordered dense nanogels, which may be caused by microwave heating and ultrasonic treatment of EWP powder.

### 2.4. Stability Analysis of P-EWP-CBEO

#### 2.4.1. Thermal Stability

**Figure 5:**
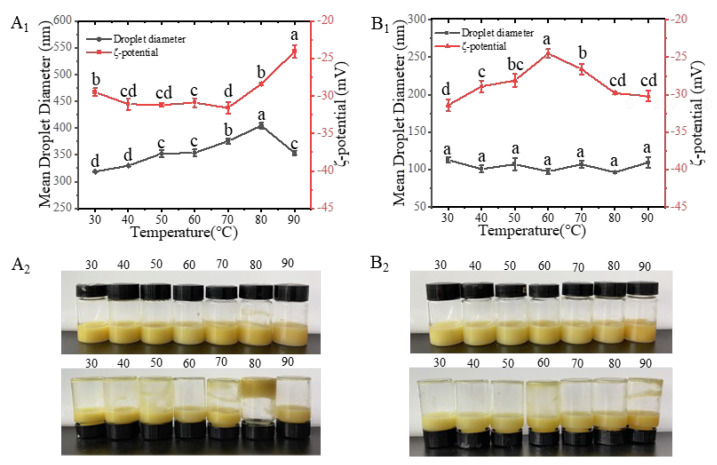
Effect of different temperatures (30–90 °C) on the particle size and zeta potential of EWP-CBEO (**A**) and P-EWP-CBEO (**B**). **A_1_** and **B_1_** represent the particle size and Zeta-potential maps of EWP-CBEO and P-EWP-CBEO; **A_2_** and **B_2_** represent the morphology of EWP-CBEO and P-EWP-CBEO, respectively. Broken lines with different lower-case letters within the same panel are significantly different at *p* < 0.05 level according to Duncan’s multiple range test.

It has been an established fact that droplet aggregation is strongly dependent on heating conditions and ionic strength [32]. Heat treatment is generally used to ensure microbiological safety and is often a method of processing food. In addition, heat treatment may also be an effective method to manipulate protein functions, especially the interfacial behavior of proteins in colloidal systems [33]. In this study, the visual observation of microscopic particle size and zeta-potential analysis of heat-treated EWP-CBEO nanogels and P-EWP-CBEO nanogels (Figure 5). During the treatment at 90 °C, the particle size of the sample increased and then decreased, reaching a maximum at 80 °C and a large number of nanogels aggregated and coagulated, indicating that heat treatment had a great impact on the stability of EWP-CBEO nanogels. Compared with EWP-CBEO, the particle size of P-EWP-CBEO nanogels did not fluctuate greatly at 30–90 °C, indicating that heating does not have a larger effect on the stability of P-EWP-CBEO nanogels. These findings are in agreement with the results reported by Teo et al. that nanoemulsions stabilized with whey protein did not change in stability after heating at different temperatures for 15 min between 30 °C and 90 °C. Other studies have shown that prolonged heat treatment of whey protein-stabilized emulsions induces extensive denaturation of globular proteins at the interface, leading to non-polar and thiol exposure and droplet aggregation through droplet-to-droplet interactions [34]. The P-EWP-CBEO nanogels, and since the heating time is not long and does not cause extensive protein aggregation, the zeta potential of the P-EWP-CBEO nanogels is not maintained. Similar results were also shown in a previous study reported by Lee et al. [35], who found protein-stabilized nanoemulsions were stable to heat-treated oil droplet aggregation due to strong electrostatic repulsion between whey protein-stabilized nanoemulsions.

#### 2.4.2. Ionic Strength

**Figure 6:**
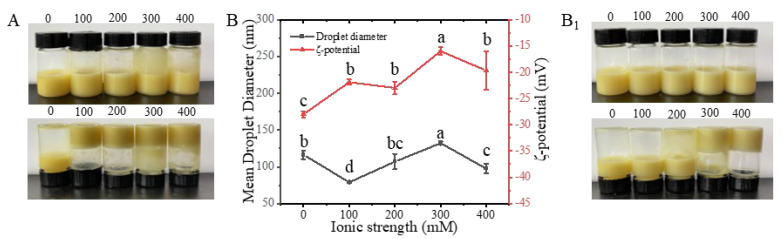
Effect of different ionic strengths on the particle size and zeta-potential of EWP-CBEO and P-EWP-CBEO nanogels. Samples were measured after being placed for 12 h at different ionic strengths (0–400 mM) for 30 min; EWP-CBEO (**A**) and P-EWP-CBEO (**B**) represent sample morphology, respectively. Samsamples were left for 12 h after 30 min at different ionic intensities (0–400 mM); (**B**): particle size and Zeta-potential map of the P-EWP-CBO; EWP-CBO (**A**) and P-EWP-CBO (**B1**) represent the morphology of the samples. Different superscript letters indicate statistically significant differences (*p* < 0.05).

P-EWP-CBEO nanogels were morphologically characterized, and their particle size was measured in relation to ionic strength (NaCl). As shown in Figure 6, the freshly prepared EWP-CBEO and P-EWP-CBEO nanogels exhibited good ocular morphology, and no aggregation, creaming, or delamination was observed. When NaCl with distinctive ionic strength (100–400 mM) was added to EWP-CBEO and P-EWP-CBEO nanogels, there were obvious differences between the two samples. For EWP-CBEO nanogels, adding NaCl with distinct ionic strength, it can be seen from Figure 6A that the samples have a large degree of aggregation, and this degree of aggregation may be due to protein aggregation at higher salt concentrations [36]. For P-EWP-CBEO nanogels, with the increase of NaCl ionic strength, a certain degree of aggregation or intermolecular approach appeared in 300–400 mM, but the surface hardness of the sample was significantly weaker than that of the EWP-CBEO nanogels. The particle size and potential diagram of the measured P-EWP-CBEO nanogels are shown in Figure 6B_1_, from which it can be seen that the P-EWP-CBEO nanogels have a certain degree of aggregation. The particle size of the egg does not change much. As a result, electrostatic repulsion is reduced, and aggregation is more likely because protein particles smaller than 30 mV easily lead to protein aggregation [37]. Therefore, in summary, P-EWP-CBEO nanogels are more stable than EWP-CBEO nanogels at different ionic strengths (0–200 mM), and phosphorylation modification improves the ionic strength stability of EWP-CBEO nanogels to a certain extent.

### 2.5. Antibacterial Activity of P-EWP-CBEO

The MIC value represents the antibacterial and bactericidal effects; as the MIC value decreases, the more effective the test material becomes as an antibacterial and bactericidal agent [9]. As shown in Table 3, the cinnamon essential oil has an effective inhibitory effect on *E. coli*, *S. aureus*, and *L. monocytogenes*, with the MIC values being 0.5, 1, and 1 mg/mL, respectively. The results showed that the antibacterial effect of CBEO on *E. coli* was higher than that of *S. aureus* and *L. monocytogenes*. The reason may be that *S. aureus* and *L. monocytogenes* are Gram-positive bacteria with thicker peptidoglycan in the cell wall and teichoic acid; *E. coli* is a Gram-negative bacterium with thin peptidoglycan and no teichoic acid in the cell wall [38]. CBEO exerts a bacteriostatic effect by destroying the cell wall, cell membrane, and DNA of microorganisms [39]. In addition, it can be seen from Table 3 that compared with CBEO, EWP-CBEO nanogels did not improve the antibacterial ability of cinnamon essential oil, but the MIC values of P-EWP-CBEO nanogels on S. aureus and *L. monocytogenes* lower by a factor of two (0.5 mg/mL), which may be attributed to the fact that CBEO is encapsulated in P-EWP-CBEO nanogels with remarkable improvement in the solubility, thereby effectively releasing antibacterial active substances into the medium [40].

The inhibition activity of CBEO, EWP-CBEO, and P-EWP-CBEO nanogels against *E. coli*, *S. aureus*, and *L. monocytogenes* was evaluated by inhibition circles as shown in Table 4. The larger the inhibition circle, the better the inhibition effect (Khalaf, Gouda, & Taleb, 2024). Initially, CBEO, EWP-CBEO, and P-EWP-CBEO nanogels all produced significant inhibition circles against the tested bacteria, with P-EWP-CBEO showing the highest inhibition circles, followed by CBEO and EWP-CBEO. Specifically, after incubation for 12 h, inhibition circles of P-EWP-CBEO nanogels against *E. coli*, *S. aureus*, and *L. monocytogenes* were 22.75, 21.20, and 19.63 mm, respectively. The diameter of the CBEO and EWP-CBEO inhibition zones decreased with increasing incubation time. In particular, CBEO did not produce clear inhibition circles against the three bacteria tested at 72 h. However, the size of the inhibition circles of P-EWP-CBEO against the three bacteria remained moderately inhibitory after 72 h. The diameters of the inhibition circles of P-EWP-CBEO against *E. coli*, *S. aureus*, and *L. monocytogenes* were 17.10, 13.25, and 15.33 mm, respectively. The single most striking observation to emerge from the data comparison was that P-EWP-CBEO has a longer-term antibacterial effect than natural CBEO and EWP-CBEO. This finding is consistent with a study where *S. aureus* was more susceptible to CBEO than *E. coli*. [41]. The antibacterial activity of CBEO, EWP-CBEO, and P-EWP-CBEO decreased significantly with increasing storage time, with P-EWP-CBEO showing stronger and longer-lasting antibacterial properties than CBEO and EWP-CBEO. According to the study, this phenomenon can be attributed to the volatilization of the unstable inhibitory component of CBEO over time [42]. In this study, the volatile components of CBEO were protected by proteins, and it was found that these components were lost slowly and reduced after nanosizing. As a result, strong long-term antimicrobial properties of CBEO are retained over time.

**Figure 7:**
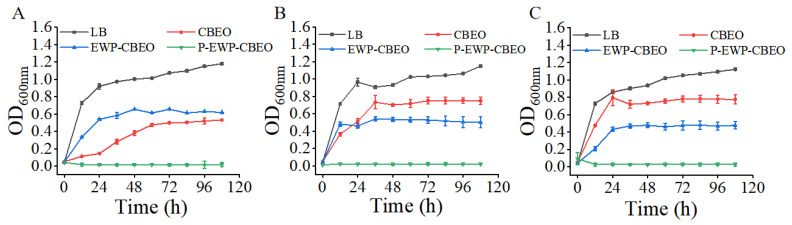
Effects of LB, CBEO, EWP-CBEO, and P-EWP-CBEO nanogels on the time growth curves of *Escherichia coli* (**A**), *Staphylococcus aureus* (**B**), and *Listeria monocytogenes* (**C**).

A time growth curve was performed to compare the antibacterial activity of CBEO, EWP-CBEO, and P-EWP-CBEO nanogels. As shown in Figure 7, the rate of bacterial proliferation in the medium was reflected by the absorbance value (Fuentes, Jofré, & Tortella, 2024). When the initial inoculation amount was 10^7^ CFU/mL, compared with free CBEO, the antibacterial ability of EWP-CBEO nanogels against the three strains significantly increased (*p* < 0.05). Precisely, the bacteriostatic ability of free CBEO on *E. coli* was stronger than that of EWP-CBEO nanogels during storage for 108 h; the bacteriostatic ability of free CBEO on *S. aureus* before storage for 24 h was stronger than that of EWP-CBEO nanogels; after 24 h of storage, the bacteriostatic ability of free CBEO was weaker than that of EWP-CBEO nanogels; the bacteriostatic ability of free CBEO to *L. monocytogenes* was weaker than that of EWP-CBEO nanogels within 108 h. Referring to the research results of Liu et al., each type of bacteria responds differently to Eos [43]. In addition, the antibacterial effect of P-EWP-CBEO nanogels on the three strains was significantly better than that of other treatment groups. The results showed that phosphorylation modification could significantly improve the antibacterial activity of EWP-CBEO nanogels, and P-EWP-CBEO nanogels completely inhibited the growth of pathogenic bacteria within 108 h. This may be because the phosphorylation-modified EWP-CBEO nanogels increased the encapsulation efficiency, increased the solubility of CBEO, and inhibited the volatilization of CBEO in the P-EWP-CBEO nanogels.

## 3. Conclusions

In this study, the phosphorylated egg white protein-cinnamon bark essential oil nanogels were prepared successfully. The FTIR spectroscopy, DSC, SEM, and TEM confirmed the presence of CBEO and STP in the P-EWP-CBEO nanogel system. The colloidal stability test results showed that, compared with EWP-CBEO nanogels, P-EWP-CBEO nanogels had stronger tolerance to temperature and ionic strength, indicating that phosphoric acid chemical modification can effectively enhance the environmental adaptability of EWP-CBEO nanogels. The results of antibacterial experiments showed that the encapsulation of protein nanogels significantly improved the antibacterial capability of CBEO, enabling longer residence time through sustained release and thus more effective bacterial killing. At the same time, protein nanogels have good dispersion, keeping CBEO constant and controllable release, so phosphorylation modification effectively enhances the antibacterial activity and long-term effects of EWP-CBEO nanogels on *E. coli*, *S. aureus*, and L. *monocytogenes*. 

## 4. Materials and Methods

### 4.1. Chemicals and Materials

EWP was purchased from Anhui Rongda Food Co., Ltd. (Xuancheng, Anhui, China). CBEO (99%) and BCA protein quantification kit were purchased from Shenggong Bioengineering (Shanghai) Co., Ltd. (Shanghai, China).

### 4.2. Bacterial Strains

*S. aureus* CICC 21600, *E. coli* CICC 10664, and *L. monocytogenes* ATCC 1911 were purchased from the China Industrial Microorganism Collection and Management Center. Strains were streaked on LB agar plates and placed in a 37 °C incubator for 24 h. A single colony was picked from the plate, and a 0.5 mL unit suspension was prepared by Mew’s turbidimetry (1.5 × 10^8^ CFU/mL), diluted with sterile saline to the desired concentration of bacterial suspension (10^5^–10^6^ CFU/mL). 

### 4.3. Preparation of P-EWP-CBEO Nanogels

**Figure 8:**
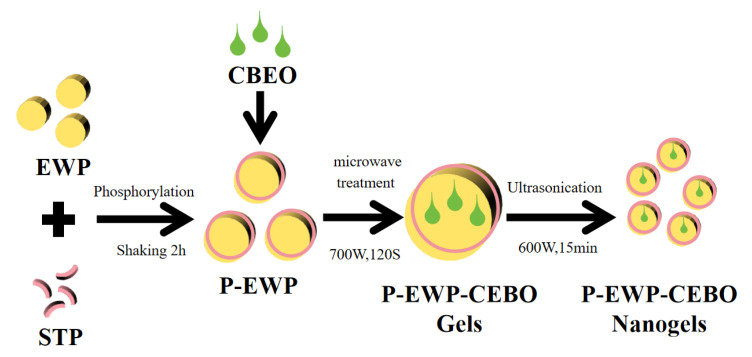
Flow chart of P-EWP-CBEO nanogel preparation.

The preparation of P-EWP-CBEO nanogels was conducted using the same procedure as our previous work [11]. Briefly, EWP was fully homogenized in distilled water and incubated at room temperature for 10 min. The mixture was centrifuged for 10 min, and the supernatant was collected. STP was added to the EWP solution at a concentration of 30 g/L. After incubation with a shake for 2 h, CBEO (dissolved in ethanol) was added, incubated, and stirred for another 3 h. After which, the solution was subjected to intermittent microwave treatment at 700 W for 120 s at 24 s intervals, followed by ultrasonic homogenization at 600 W for 15 min at 3 s on/off pulse using an ultrasonic disintegrator. The resulting mixture was dialyzed in deionized water at 4 °C for 24 h to remove redundant phosphate, pre-frozen at −80 °C for 4 h, and then freeze-dried in a freeze dryer for approximately 48 h. Freeze-dried samples were subsequently ground into powder, sifted through a mesh sieve, and stored in a seal dryer at 4 °C until later assessment. EWP-CBEO nanogels without CBEO were prepared by the above method and served as a control. As shown in Figure 8.

### 4.4. Particle Size and Zeta Potential of P-EWP-CBEO Nanogels

The particle size, zeta potential, and particle dispersion index (PDI) of P-EWP-CBEO nanogels in four replicates were measured by using Zetasizer Nano ZS (Malvern Instruments, UK). The temperature was set to 25 °C and the scattering angle was set to 90°. The experiments were conducted in three steps; each measurement was composed of 12 independent runs lasting 10 s each, followed by 120 s of equilibration. 

### 4.5. Encapsulation Efficiency (EE) of P-EWP-CBEO Nanogels

A spectrophotometric method reported by Rao [44] was adopted with some modifications to determine the CBEO content. The absorbance of CBEO standard solutions was measured at 287 nm with various concentrations of ethanol absolute (0.5, 1, 1.5, 2, 2.5, and 3 mL/L) by using ethanol absolute as the blank control. CBEO concentration and absorbance were the abscissa and ordinate, respectively. The obtained standard calibration curve equation was as follows: Y = 0.1171x + 0.0127, R^2^ = 0.999.

A 0.5 g of freeze-dried pellets was fully mixed with 10 mL of ethanol and incubated in a shaker. At 25 °C for 5 min, ultrasonically extracted for 60 min, and centrifuged for 10 min to obtain the total oil. Absorbance values of all the solutions were measured at 287 nm, and CBEO content was determined based on the standard calibration curve. The EE was calculated as follows:

EE% = (W_1_ − W_2_)/W_0_ × 100

In this equation, W_1_ is the mass of total oil in particles, W_2_ is the mass of oil on particle surfaces, and W_0_ is the mass of oil added to the system.

### 4.6. Intermolecular Force Assays of P-EWP-CBEO Nanogels

Protein nanogel dispersions (10 g/L) were mixed with equal volumes of reagents (10 g/L) SDS, 10 M urea, and 0.06 M DTT, respectively [45]. After 20 min of warming, the particle diameters were measured using Zetasizer Nano ZS (Malvern Instruments, Malvern, UK). For hydrated particle size measurements, the protein dispersion was diluted in deionized water, filtered through 0.22 μm filter membranes, and the filtrate was taken as the test sample. Three parallels were made for each sample, and 12 consecutive readings were taken for each measurement. The change in particle size can reveal the intermolecular forces of protein nanogels.

### 4.7. Structural Characterization of P-EWP-CBEO

#### 4.7.1. Fourier Transform Infrared Spectroscopy (FTIR)

The spectra of EWP-CBEO and P-EWP-CBEO nanogels were obtained by infrared spectrometer (Nexus 470, Nicolet, Madison, WI, USA). Protein powder was mixed with dried KBr powder at a ratio of 1:150 and then pressed into pieces. Additionally, both samples had spectra between 400 and 4000 cm^−1^ averaged over 32 scans and with a 4 cm^−1^ resolution on average. Furthermore, the background of each sample was scanned using KBr.

#### 4.7.2. Differential Scanning Calorimetry (DSC)

The thermal stability of EWP, CBEO, EWP-CBEO, and P-EWP-CBEO was determined using Q200M thermal analysis (TA Instruments, New Castle, DE, USA). The sample chamber was filled with all materials (6 mg) and heated until they were dissolved under a nitrogen atmosphere from 25 °C to 350 °C at a ramp rate of 10 °C/min with a flow rate of 20 mL/min.

#### 4.7.3. Scanning Electron Microscopy (SEM)

Reference [46] was made to observe the microstructure of EWP-CBEO and P-EWP-CBEO nanogels using an SEM (Gemini SEM 300, Carl Zeiss, Germany). A double-sided conductive carbon tape was attached to the sample stage of the scanning electron microscope, and then gold was sprayed on the surface of the material using a gold sprayer. The accelerating voltage was set to 20 kV. All steps were performed at room temperature.

### 4.8. Stability Analysis of P-EWP-CBEO

The method reported by Pumival et al. was adopted with slight modifications to evaluate the effect of environmental stress on the stability of EWP-CBEO and P-EWP-CBEO nanogels [47].

#### 4.8.1. Thermal Stability

Incubating the newly prepared nanoparticle dispersions in a water bath for 30 min (30–90 °C) required two dilutions in double-distilled water. A particle size distribution was analyzed after the samples were cooled to ambient temperature overnight.

#### 4.8.2. Ionic Strength

Using freshly prepared nanoparticle dispersions, solution of NaCl (0 to 400 mM) was mixed with the same volume of nanoparticle dispersions. Afterward, particle size distributions were measured on samples that were stored overnight at room temperature.

### 4.9. Antibacterial Assays

Oxford cup plate assays were used to determine the zones of inhibition for CBEO, EWP-CBEO, and P-EWP-CBEO nanogels. 0.2 mL of the test bacteria suspension with a concentration of 10^6^–10^7^ CFU/mL was taken and spread evenly on the surface of the sterilized medium, and 4 bacteria were placed in the center of the plate. Bacteria Oxford cup, respectively, adds 100 μL of CBEO, EWP-CBEO, and P-EWP-CBEO nanogels into the Oxford cup, measuring the diameter of the inhibition zone at 12, 24, 36, 48, 60, and 72 h. A sterile LB liquid medium was used as a control. *P. fluorescens* isolate ZX was maintained in our laboratory. It was grown on a nutrient broth agar (NA) plate at 30 °C for 2 d, and then a single colony was transferred, inoculated in NB, and cultured on a rotary shaker (200 r/min) at 30 °C overnight. Then, the bacterial suspension (*P. fluorescens* ZX in sterile distilled water (SDW)) and bacterial fluid (*P. fluorescens* ZX in NB medium) at the concentration of 1 × 10^8^ colony-forming units (CFU)/mL were prepared.

The MIC of CBEO, EWP-CBEO, and P-EWP-CBEO nanogels was determined by the micro broth dilution method, as previously described with some modifications [48]. Each sample was precisely measured with a uniform concentration of CBEO. After UV sterilization, 100 µL of LB liquid medium was added to each well, and then 100 µL of bacteriostatic agent was added to the first well of each row, and then pipet and mixed thoroughly with a pipette tip. Pipette 100 µL to the second hole, pipette to the 10th hole in turn, then add 100 µL of the bacterial solution (10^7^ CFU/mL) to the 1–9 holes and the 11th hole and add 100 µL of sterile liquid LB to the 10th hole. Make the CBEO concentration in wells 1–9 to 8, 4, 2, 1, 0.5, 0.25, 0.125, 0.0625, and 0.03125 g/L, respectively. Place the plate in a 37 °C constant temperature incubator for 24 h. The microplate reader (Infinite F50, Shanghai) measures the optical density [2] 600 nm value of each well and performs three parallel experiments for each concentration gradient.

An analysis of time-killing kinetics was conducted to determine the antimicrobial efficiency of CBEO against *E. coli*, *S. aureus*, and *Listeria monocytogenes,* and bacterial growth curves were measured in 96-well polystyrene microtiter plates for all bacterial strains as previously reported with slight modifications [49]. Measurements were carried out using an enzyme marker. Fresh liquid cultures of 2% of different bacterial solutions were incubated at 37 °C for 12 h. The final concentration of the bacterial solution was adjusted to 10^7^ CFU/mL, and the same final concentrations of CBEO, EWP-CBEO, and P-EWP-CBEO nanogels were added to the bacterial suspensions and incubated on a shaker at 37 °C. The control group was Luria–Bertani medium (LB), and absorbance was measured at 600 nm every 2 h. The growth curve was constructed using the incubation time as the horizontal axis and the OD value as the vertical axis. Growth curves were obtained for growth in NB medium at 30 °C with shaking in a rotary shaker at 200 r/min. The culture turbidity was measured every 2 h using a nephelometer. Then growth curves were fitted by the logistic equation, and the model parameters were calculated by differential calculation to obtain specific growth rate curves.

### 4.10. Statistical Analysis

Each experiment was conducted twice using SPSS, with each treatment in triplicate. All experimental data were expressed as mean ± standard deviation (X ± SD). Statistically significant differences between different treatments were assessed using Duncan’s multiple range test at *p* < 0.05.

## Figures and Tables

**Table 1:** Average particle size, PDI, and Zeta potential, and encapsulation efficiency (EE) of loaded cinnamon bark essential oil nanoparticles.

Sample Name	Particle Size/nm	PDI	Zeta/mV	EE/%
EWP-CBEO	378.8 ± 28.38 b	0.418 ± 0.102 b	−24.3 ± 0.416 a	78.34 ± 2.88% a
P-EWP-CBEO	133.55 ± 0.836 a	0.265 ± 0.006 a	−35.4 ± 0.057 b	93.79 ± 1.93% b

Each value is the mean of three replicates ± standard error. Values in a column followed by a different letter are significantly different according to Duncan’s multiple range test at *p* < 0.05 level.

**Table 2:** Thermodynamic parameters of P-EWP-CBEO, EWP-CBEO, CBEO, and N-EWP.

Samples	*T_O_* (°C)	*T_d_* (°C)	*T_p_* (°C)	△*H* (J/g)
P-EWP-CBEO	53.65	105.68	82.08	43.876
EWP-CBEO	52.66	106.68	81.12	84.107
CBEO	260.77	273.43	267.02	217.123
N-EWP	70.53	104.34	87.82	201.965

**Table 3:** Minimum inhibitory concentration (mg/mL) of CBEO, EWP-CBEO nanogels, and P-EWP-CBEO nanogels against *E. coli*, *S. aureus*, and *L. monocytogenes*.

Strain	CBEO (mg/mL)	EWP-CBEO	P-EWP-CBEO
*Escherichia coli*	0.5 b	1.0 a	0.5 b
*Staphylococcus aureus*	1.0 a	1.0 a	0.5 b
*Listeria monocytogenes*	1.0 a	1.0 a	0.5 b

Each value is the mean of three replicates ± standard error. Values in a column followed by a different letter are significantly different according to Duncan’s multiple range test at *p* < 0.05 level.

**Table 4:** Inhibition zone diameter of CBEO, EWP-CBEO nanogels, and P-EWP-CBO nanogels against test microorganisms.

Sample	Inhibition Zone Diameter (mm), Disk Diameter 6.0 mm Was Included					
	12 h	24 h	36 h	48 h	60 h	72 h
	*Escherichia coli*					
CBEO	20.38 ± 3.33 a	20.38 ± 3.47 b	20.83 ± 2.02 ab	20.50 ± 2.65 ab	17.83 ± 0.29 b	11.83 ± 1.44 c
EWP-CBEO	18.50 ± 2.31 b	18.50 ± 3.47 b	18.83 ± 2.02 b	18.00 ± 2.29 b	18.67 ± 1.53 b	15.27 ± 0.25 b
P-EWP-CBEO	22.75 ± 2.22 a	21.13 ± 1.60 a	21.50 ± 2.00 a	22.50 ± 2.78 a	21.33 ± 0.58 a	17.10 ± 1.53 a
	*Staphylococcus aureus*					
CBEO	16.88 ± 1.25 b	16.13 ± 0.63 b	16.50 ± 0.91 b	15.50 ± 0.71 b	13.75 ± 2.06 b	10.83 ± 1.61 b
EWP-CBEO	17.05 ± 0.42 b	16.13 ± 0.63 b	16.25 ± 0.96 b	14.75 ± 0.50 b	12.88 ± 1.03 b	10.38 ± 0.48 b
P-EWP-CBEO	21.20 ± 0.87 a	21.38 ± 1.37 a	19.75 ± 2.63 a	20.00 ± 2.94 a	16.00 ± 2.31 a	13.25 ± 1.50 a
	*Listeria monocytogenes*					
CBEO	15.75 ± 0.65 b	15.75 ± 0.65 b	15.17 ± 1.26 b	13.33 ± 0.29 c	11.07 ± 0.29 c	10.50 ± 1.00 c
EWP-CBEO	14.50 ± 0.77 b	15.25 ± 0.65 b	15.00 ± 0.50 b	14.50 ± 0.20 b	13.17 ± 0.29 b	13.67 ± 0.29 b
P-EWP-CBEO	19.63 ± 1.43 a	18.25 ± 0.65 a	17.67 ± 0.76 a	15.83 ± 2.02 a	15.17 ± 0.29 a	15.33 ± 0.76 a

Each value is the mean of three replicates ± standard error. Values in a column followed by a different letter are significantly different according to Duncan’s multiple range test at *p* < 0.05 level.

## Data Availability

The data presented in this study are openly available in article.

## Supplementary Materials

The following supporting information can be downloaded at: https://www.mdpi.com/article/10.3390/gels11010012/s1, Figure S1: Changes in the intermolecular forces of P-EWP-CBO colloidal particles during phosphorylation. Different letters indicate significant differences between the same sample (*p* < 0.05).
