# Supplementary material for: Contribution of Phosphorylation Modification to Stability and Antibacterial Activity of Egg White Protein Nanogels Loaded with Cinnamon Bark Essential Oil
Source: Gels. 2024 Dec 27;11(1):12. doi: 10.3390/gels11010012 (PMC11765320; doi:10.3390/gels11010012)
Supplement: Supplementary file 1 [file gels-11-00012-s001.zip › gels-3374082-supplementary.pdf]

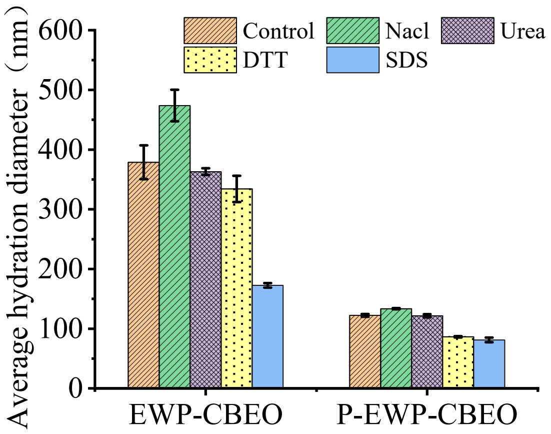

Figure S1. Changes in the intermolecular forces of P-EWP-CBO colloidal particles during phosphorylation. Different letters indicate significant differences between the same sample ($p < 0.05$).
